# Low birth weight and macrosomia in Tigray, Northern Ethiopia: who are the mothers at risk?

**DOI:** 10.1186/s12887-017-0901-1

**Published:** 2017-06-12

**Authors:** Hayelom Gebrekirstos Mengesha, Alem Desta Wuneh, Berhe Weldearegawi, Divya L. Selvakumar

**Affiliations:** 1 College of Medicine and Health Sciences, Adigrat University, Adigrat, Ethiopia; 2 School of Public Health, Mekelle University, Mekelle, Ethiopia; 3 School of Public Health, Montgomery College, Rockville, MD USA

**Keywords:** Low birth weight, Macrosomia, Multinomial regression, Northern Ethiopia, Tigray

## Abstract

**Background:**

Infant birth weight, which is classified into low birth weight, normal birth weight and macrosomia, is associated with short and long-term health consequences, such as neonatal mortality and chronic disease in life. Macrosomia and low birth weight are double burden problems in developing counties, such as Ethiopia, but the paucity of evidence has made it difficult to assess the extent of this situation. As a result there has been inconsistency in the reported prevalence of low birth weight and macrosomia in Ethiopia. This study aimed to determine the incidence and predictors of low birth weight and macrosomia in Tigray, Northern Ethiopia.

**Method:**

We conducted a cross-sectional survey among a cohort of 1152 neonates delivered in Tigray Region at randomly selected hospitals between April and July 2014. We used the birth weight category described previously as an outcome variable. Data were collected using structured questionnaire by midwives. We entered and analyzed data using STATA™ Version 11.0. Data were described using a frequency, percentage, relative risk ratio, and 95% confidence interval. Multinomial logistic regression was conducted to identify independent predictors of low birth weight and macrosomia.

**Result:**

In this study, we found a 10.5% and 6.68% incidence of low birth weight and macrosomia, respectively. Seventy (57.8%) of all low birth weight neonates were term births. The predictors for low birth weight were: early marriage (<18 year) (RRR: 0.59, CI: 0.35–0.97); rural residence (RRR: 0.53, CI: 0.32–0.9); prematurity (RRR: 15.4, CI: 9.18–25.9); no antenatal follow-up (RRR: 6.78, CI: 2.39–19.25); and female sex (RRR: 1.77, CI: 1.13–2.77). Predictors for macrosomia were: female gender (RRR: 0.58, CI: 0.35–0.9); high body mass index (RRR: 5.0, CI: 1.56–16); post-maturity (RRR: 2.23, CI: 1.06–4.6); and no maternal complication (RRR: 0.46, CI: 0.27–0.8).

**Conclusion:**

In this study, we found gestational age and gender of the neonate to be common risk factors for both low birth weight and macrosomia. Strengthening antenatal follow up, prevention of pre and post maturity, controlling body mass index, and improving socioeconomic status of mothers are recommendations to prevent the double burden (low birth weight and macrosomia) and associated short and long-term consequences.

## Background

Birth weight is an important indicator in the prediction of short- and long-term health outcomes [1]. Based on birth weight, the World Health Organization (WHO) classifies infants as Low Birth Weight(LBW) (<2500 g), Normal birth weight (2500–4000), and Macrosomia (≥4000 g) [2, 3]. Evidence shows that pregnancies with macrosomic infants are associated with increased risk of complications for both the neonates and their mothers [4–6]. Macrosomia is associated with increased risk of perinatal asphyxia, death, and shoulder dystocia. Similarly, mothers with macrosomic pregnancies are at an increased risk of caesarean section, prolonged labor, abnormal hemorrhage, perineal trauma and adverse perinatal outcomes [4–6]. The incidence of fetal macrosomia significantly varies across different geographic regions, with frequency ranging from 0.5% to 15% in 23 developing countries in Africa, Asia, and Latin America [6]. In developing countries the prevalence of LBW is estimated to be 19%, in comparison to between 5% and 7% in developed countries, [7]. In sub-Saharan Africa, the rate of LBW has been reported between 12 and 17% [8–10].

Studies also showed macrosomia to be associated with long-term consequences including obesity, diabetes, and heart disease [11–15]. LBW is a significant determinant of infant and childhood morbidity, particularly of neuro-development impairments, such as mental retardation and learning disabilities. It is also closely associated with fetal and neonatal mortality and morbidity, inhibited growth, cognitive delays, and chronic diseases later in life [16]. Hence, both macrosomia and LBW are associated with long-term consequences, which may lead to economic and social burdens unless efforts are undertaken to modify/address the major risk factors.

Several maternal characteristics, such as prior history of diabetes, prolonged gestation, multiparty, older maternal age, short height, high body mass index, and significant weight gain during pregnancy are associated with an increased risk of macrosomia [6, 17–19]. Due to a rapid increase in the prevalence of overweight and obese conditions of the reproductive age population, macrosomia and its complications are rapidly increasing in developing countries [6]. However, in Ethiopia, there is no evidence on the levels of macrosomia and complications resulting from it.

There is evidence that the risk factors for LBW, such as high risk pregnancy, extremes of age at time of pregnancy, anemia, pregnancy-induced hypertension, and poor nutritional status, are widely reported in Sub-Saharan Africa including Ethiopia [8–10, 20, 21]. Though there is not sufficient evidence regarding the magnitude of LBW, some studies in Ethiopia have reported that the incidence may be as high as one LBW in every 10 live births [9, 22].

Findings from recent studies in Ethiopia have shown that despite the decline in infant and child mortality, neonatal mortality remains unacceptably high [22, 23]. Despite the significant improvement in health and health related indicators over the last decade, Ethiopia still bears one of the highest burdens of maternal mortality [23, 24]. Of note, a recent study in eastern Ethiopia reported that one in every five pregnant women were malnourished [25]. It is also well established that LBW is the significant contributor for neonatal and infant deaths [26]. It is in this context, that the imperative to investigate the levels and underlying risk factors of both LBW and macrosomia arises in order to inform future policy and interventions to reduce maternal and neonatal morbidity and mortality.

The present study aims to measure the levels of LBW and macrosomia among women in Tigray region. In addition, the study identifies maternal and obstetric characteristics associated with the risk of LBW, and macrosomia in this setting in an effort to identify potentially modifiable factors in order to mitigate their short and long-term consequences.

## Method

### Study design

This study is a part of a large prospective observational study undertaken in one of the nine administrative regions of Ethiopia to investigate pregnancy outcome and neonatal survival. The current study is a cross-sectional survey of 1162 neonates-maternal dyads delivered in select hospitals between April and July 2014 in Tigray, Ethiopia. Details of sample size calculation and sampling procedure have been published previously [27].

### Study setting and population

This study was conducted in seven randomly selected public hospitals in Tigray region, northern Ethiopia. Tigray Region is one of the 11 administrative states in Ethiopia with a total population of 5,055,999 (49.2% male and 50.8% female). The Ethiopian health system is structured into a three-tier system with the primary care level comprised of health posts, health centers and primary hospitals, the secondary care level including general hospitals, and the tertiary care level of specialized hospitals. In Tigray region there are 15 public general hospitals, 1 specialized referral hospital and 20 primary hospitals, 204 health centers and 712 health posts. All general hospitals and specialized referral hospital provide comprehensive maternal and newborn services, emergency and elective caesarean sections. This study was conducted in the specialized referral hospital, and six general hospitals which were randomly selected from the 16 hospitals of the region by lottery method [28].

### Data collection and quality assurance

Data were collected using a pre-tested structured questionnaire on 5% of the sample size to improve clarity and modify the data collection instruments and a checklist adapted from the WHO standard verbal neonatal autopsy questionnaires [29]. The questionnaire was initially developed in English and translated to local language (i.e., Tigrigna). Two degree-prepared midwives, who were fluent Tigrigna speakers, were recruited for data collection. The data collectors interviewed all mothers who had live births at the selected hospitals. This interview was done within the first 6-h after delivery. In addition, clinical information, including maternal complications, birth weight, gestational age and any relevant medical diagnosis, was extracted through assessment of the neonate and mother. Prospective data collected including sociodemographic, neonatal, maternal, and health service-related characteristics.

### Inclusion and exclusion criteria

Women, who experienced live births in the study hospitals from April –July, 2014 were included in the study. Mothers who were unable to speak and mothers with psychiatric illnesses were excluded from the study.

### Assessment and definition of variables

Considering previous evidence and the nature of our study, which includes both clinical and sociodemographic characteristics, the independent variables were broadly categorized as distal, intermediate, and proximal. Distal variables included socioeconomic and demographic factors such as: reported income [low (<500 ETB), medium (500–2000 ETB) and rich (>2000ETB)]; residence [urban and rural]; age at first marriage [below age of 18, 18 years and above]. Intermediate factors related to maternal, neonatal, and health services included: maternal factors, such as body mass index (BMI) [underweight (BMI less than 18.5), normal (BMI 18.5–24.99), overweight (BMI >25.0)]; fertility (number of live children) [primiparae, 2–4 and multiparty (≥5)]; antenatal follow up is defined as care or supervision given to a pregnant, parturient, peurperal woman so as to enable her to pass through the dangers of pregnancy with least possible risk. It was categorized into [“yes” if the mother had at least one antenatal follow up; “no” if the mother never had antenatal follow up]; neonatal factors such as birth weight [measured using a standard beam balance within one hour of delivery], gender [female/male], gestational age [measured by using last menstrual period and/or ultrasound and categorized as preterm (<259 days), term (259–293 days) and post- term birth (≥294 days]

Diagnosed medical disease was assessed using “yes” if the mother had a medical diagnosis (i.e., HIV/AIDS, hypertension, or diabetes mellitus), either during the delivery or before delivery and “no” if the mother did not have such a diagnosis.

Proximal variables included maternal complication (i.e., obstetric hemorrhage, puerperal sepsis and pyrexia, prolonged labour, (pre)eclampsia, malpresentation or malposition, premature rupture of membranes, cord prolapse, obstructed labour, cephalo-pelvic disproportion, emergency caesarean section, and retained placenta). Categorized as “yes” or “no”.

### Data analysis and management

The distribution of maternal and neonatal characteristics by type of pregnancy outcome was summarized using frequency distribution, with prevalence of LBW and macrosomia were also reported. Categorical data was described using frequency and percentage. Birth weight was analyzed by categorizing into three outcome variables - LBW, normal birth weight (base) and macrosomia, which were considered as nominal variables.

To identify the predictors of LBW and macrosomia, we used multinomial logistic regression. A bivariable multinomial logistic regression was performed using the crude risk ratio of regression, and variables with likelihood ratio test *P*-values of less than 0.05 were retained in the multiple regression models. Multivariable multinomial logistic regression was used to estimate the relative risk ratio (RRR) and corresponding 95% confidence interval (CI) of the association between birth weight and predictors at *P* < 0.05. Interaction between predictor variables was tested at a significance level *P* < 0.05 by including an interaction term in the multivariate model. Potential predictors were categorized into three hierarchies: 1) proximal factors 2) intermediate factors (such as maternal, neonatal and health service factors) and 3) distal factors (such as sociodemographic and economic variables). Data was entered, cleaned, and analyzed using STATA™ version 11.0 statistical packages.

## Result

### Sociodemographic characteristics of participants

Responses were obtained from all participants (*n* = 1162); however, ten (0.96%) observations were excluded from the analysis due to incompleteness. The mean current age of mothers was 26.6 ± 5.4. Nearly half 55(45%) of the mothers of LBW babies were married before age 18, while almost two-third 50(64.8%) of mothers of macrosomic babies married at the age of 18 or above.

The study revealed the highest number [25(20.7%)] of LBW neonates and of macrosomia neonates [16(20.6%)] were delivered in Ayder (referral) and Suhl (Zonal) hospitals, respectively. Maternal residence indicated 755(65.5%) resided in the urban settings and the remaining numbers resided in the rural areas. Of these, the highest proportion of 62 mothers (51.2%) of LBW neonates were delivered by rural mothers, while 54 mothers (70.1%) of macrosomic neonates were from urban mothers (Table 1). Bivariable analysis using multinomial regression showed urban residence was associated with LBW (RRR: 0.46, CI: 0.3–0.67) but not associated with macrosomia (RRR: 1.1, CI: 0.68–1.89). Mothers who married first at ≥18 years old were at less risk to deliver LBW (RRR: 0.5, CI: 0.35–0.75) but maternal age was not associated with macrosomia (RRR: 0.79, CI: 0.48–1.29).

**Table 1:** Association of selected socio-demographic characteristics with low birth weight and macrosomia on neonates born in Tigray, Northern Ethiopia, April–July, 2014 (*n* = 1152)

Characteristics	Total live births		LBW	Macrosomia	LBW & Macrosomia
	Freq.	Percent	Freq (RRR, 95% CI)	Freq (RRR, 95% CI)	*P*-value
Hospital					
Ayder Referral	139	12.1	25	12	
Adwa Hospital	198	17.2	19	11	
Lemlem Karl	147	12.8	15	8	
Suhl Hospital	143	12.4	23	16	
Kahsay Abera	102	8.8	7	8	
Kidist Mariam	213	18.5	21	12	
Adigrat	210	18.2	11	10	
Residence					
Rural	397	34.5	62(ref)	23(ref)	
Urban	755	65.5	59(0.46, 0.3–0.67)	54(1.1, 0.68–1.89)	<0.001,0.61
Educational status					
Unable to read	275	23.9	50(ref)	15(ref)	
Primary	338	29.3	23(0.33, 0.2–0.56)	24(1.15, 0.59–2.25)	<0.001,0.67
Secondary	361	31.3	31(0.42, 0.26–0.7)	22(1, 0.5–1.97)	<0.001,0.99
Tertiary	178	15.5	17(0.49, 0.27–0.9)	16(1.54, 0.74–3.22)	0.019, 0.25
Income^a^					
Poor	327	32.2	40(ref)	20(ref)	ref
Medium	316	31.1	33(0.83, 0.5–1.36)	20(1.01, 0.53–1.93)	0.47,0.31
Rich	373	36.7	36(0.78, 0.5–1.25)	29(1.25, 0.69–2.27)	0.96,0.45
Age at marriage					
< 18	368	31.9	55(ref)	27(ref)	
≥ 18	784	68.1	66(0.5, 0.35–0.75)	50(0.79, 0.48–1.29)	0.001,0.35

*RRR* Relative Risk Ratio, *CI* Confidence Interval, *Ref* Reference

^a^Rich ≥ 1500, Ethiopian birr (ETB), Medium:600–1500 ETB, Poor: <600 ETB

### Prevalence of low birth weight, and macrosmonia

One in every 10 live births (10.5%) was underweight (121/1152), and 6.7% (77/1152) were macrosomic, while the remainder (954/1152) were within the normal range of birth weight. Considerable LBW babies [40% (49/121)] were pre-term births **(**Table 2).

**Table 2:** Association of select maternal and neonatal characteristics with low birth weight and macrosomia on neonates born in Tigray Region, Northern Ethiopia (*n* = 1152)

Characteristics	Total live births		LBW	Macrosomia	LBW, Macrosomia
	Freq.	Percent	Freq.(RRR,95% CI)	Freq(RRR,95% CI)	*P*-value
Birth weight					
Low birth weight	121	10.5			
Normal	954	82.8			
Macrosomia	77	6.7			
Current age					
< 20 year	95	8.2	9(ref)	6(ref)	ref
20–24 year	328	28.5	32(1.0, 0.47–2.24)	20(0.96, 0.37–2.5)	0.94,0.94
25–35 year	590	57.2	65(1.2, 0.57–2.48)	40(1.1, 0.45–2.67)	0.64,0.83
≥ 35 year	139	12.1	15(1.17, 0.49–2.8)	11(1.3, 0.46–3.65)	0.71,0.62
Number of children					
Primiparae	610	53.0	60(ref)	36(ref)	ref
2–4 children	424	36.8	39(0.94, 0.61–1.9)	32(1.29, 0.78–2.1)	0.80,0.30
Multiple(≥5)	118	10.2	22(2.16, 1.26–3.7)	9(1.47, 0.68–3.17)	0.005,0.31
Birth interval					
Not applicable	537	46.6	52(ref)	29(ref)	ref
< Two years	127	11.0	10(0.8, 0.39–1.64)	9(1.31, 0.60–2.84)	0.56,0.49
> Two years	488	42.4	59(1.32, 0.9–1.97)	39(1.57, 0.95–2.6)	0.16,0.07
Disease					
Yes	100	8.7	9(ref)	5(ref)	ref
No	1052	91.3	112(1.23, 0.6–2.5)	72(1.42, 0.56–3.6)	0.56,0.45
Maternal complications					
Yes	221	19.2	34(ref)	23(ref)	ref
No	930	80.8	86(0.52, 0.34–0.8)	54(0.48, 0.29–0.8)	0.003,0.006
Sex					
Male	610	52.9	54(ref)	50(ref)	ref
Female	542	47.1	67(1.4, 0.95–2.04)	27(0.6, 0.37–0.99)	0.082,0.046
Body mass index					
Underweight	119	10.6	14(ref)	4(ref)	ref
Normal	907	80.8	94(0.9, 0.49–1.63)	58(1.9, 0.68–5.45)	0.72,0.20
Overweight	96	8.6	12(1.25, 0.54–2.8)	15(5.48, 1.7–17.2)	0.59,0.004
Gestational age					
Preterm	93	8.1	49(13.6,8.5–22)	1(0.3, 0.23–2.21)	<0.001,0.12
Term	990	85.9	71(ref)	66(ref)	
Post term	69	6.0	1(0.2, 0.02–1.51)	10(2.22, 1.1–4.56)	0.23,0.03
Mode of delivery					
Vaginal	846	73.5	90(ref)	44(ref)	
Cesarean section	241	20.9	27(1.15, 0.72–1.8)	29(2.5,1.54–4.16)	0.54,<0.001
Instrumental	65	5.6	4(0.55, 0.2–1.56)	4(1.13, 0.4–3.27)	0.26,0.81
Antenatal follow up					
Yes	1131	98.2	113(ref)	76(ref)	
No	21	1.8	8(5.55, 2.2–13.88)	1(1.03, 0.13–8.05)	<0.001,0.975

*RRR* relative risk ratio, *CI* confidence interval, *LBW* low birth weight, *Freq*.: Frequency

#### Maternal and neonatal characteristics

Regarding maternal complication, 19.2% of the mothers had complications, of which 15% related to macrosomic births and 10.4% to LBW deliveries. With regard to mode of delivery, 241(20.92%) babies were delivered by Caesarean section and, of these, 29(12%) were macrosomic neonates and 27(11.2%) preterm. Neonates showed a 1:1.12 male to female ratio. Nearly 60% (71) of LBW babies were at term **(**Table 2 **).**

On bivariable analysis, using multinomial regression, multiparty (≥5) was associated with LBW but not with macrosomia (RR: 2.16, CI: 1.26–3.7). Four in ten LBW births (40%) was preterm, which was associated with LBW (RRR: 2.1, CI: 1.24–3.8) and post-term birth was significantly associated with macrosomia (RRR: 2.48, CI: 1.2–5.12) (Table 2).

#### Multivariate analysis

In the multivariable regression, having no antenatal follow-up, early marriage, preterm birth, rural residence, and female gender of neonate were significant predictors of LBW, while high body mass index of mothers, post-term birth, having a male neonate, and maternal complications were associated with macrosomia (Table 3).

**Table 3:** Multivariate analysis using hierarchical regression on predictors of low birth weight and macrosomia on neonates born in Tigray Region, Northern Ethiopia, April–July, 2014 (*n* = 1152)

Predictors	Model 1:		Model 2:		Model 3:	
	Proximal	Factors	Intermediate	Factors	Distal	Factors
	RRR, 95% CI LBW	RRR, CI 95% macrosomia	RRR, 95% CI LBW	RRR, 95% CI macrosomia	RRR, 95% CI LBW	RRR, 95% CI macrosomia
Body mass index						
Normal			ref	ref	ref	ref
Underweight			1.0(0.5–1.99)	1.93(0.68–5.5)	1.01(0.50–2.03)	1.9(0.67–5.48)
Overweight			1.73(0.67–4.5)	5.2(1.6–16.5)	1.9(0.73–5.05)	5(1.56–16)
Gestational age						
Term			ref	ref	ref	ref
Preterm			15.5(9.32–25.7)	0.2(0.03–2.0)	15.4(9.18–25.9)	0.26(0.35–2.0)
Post term			0.21(0.02–1.59)	2.26(1.08–4.7)	0.2(0.027–1.52)	2.23(1.06–4.6)
Antenatal(Yes ref)			6.9(2.49–19.5)	0.8(0.11–7.26)	6.78(2.39–19.2)	0.9(0.11–7.76)
Female sex(male ref)			1.7(1.1–2.68)	0.58(0.35–0.9)	1.77(1.13–2.77)	0.58(0.35–0.9)
Complication(yes ref)	0.52(0.34–0.8)	0.48(0.29–0.81)	0.74(0.44–1.23)	0.47(0.27–0.8)	0.74(0.44–1.24)	0.46(0.27–0.8)
Parity						
Priampre			ref	ref	ref	ref
Two			0.83(0.51–1.36)	1.19(0.71–2.0)	0.83(0.5–1.38)	1.21(0.72–2.0)
Multiparity (≥3)			1.65(0.86–3.18)	1.39(0.61–3.1)	1.27(0.62–2.61)	1.67(0.7–3.9)
Age at marriage						
≥18 (“<18” ref)					0.59(0.35–0.97)	0.71(0.4–1.23)
Residence(“Rural” ref)					0.53(0.32–0.9)	1.08(0.6–1.96)
Education						
Unable to read & write					ref	ref
Primary					0.53(0.27–1.01)	1.31(0.2–2.7)
Secondary					0.99(0.5–1.98)	1.39(0.6–3.2)
Tertiary					1.2(0.52–2.7)	1.85(0.74–4.6)

*ref reference, *RRR* Relative Risk Ratio, *CI* Confidence Interval, *LBW* Low Birth Weight

Mothers classified as high BMI were 5 times more likely to be to deliver macrosomic neonates than mothers in the normal BMI category (RRR: 5.0, CI: 1.56–16)). Regarding gestational age, the risk of macrosomia was twice as high in post-term neonates compared to term babies (RRR: 2.23, CI: 1.06–4.6)) and risk of LBW was fifteen times higher in pre-term neonates than in term neonates (ARR: 15.4, CI: 9.18–25.9). Female neonate were found 42% less at risk of becoming macrosomic than their counterpart male neonates (RRR: 0.58, CI: 0.35–0.9), but 1.77 times (RRR: 1.77, CI: 1.13–2.77) more likely to experience low birth weight than their male counterparts. Similarly, mothers who had no complication around birth had a 54% lower risk of delivering macrosomia (RRR: 0.46, CI: 0.27–0.8) than these mothers who had complications. Delivery complications were not significantly associated with LBW (RRR: 0.74, CI: 0.44–1.24). Concerning antenatal follow-up, with relative to normal birth weight neonates mothers who never had antenatal follow-up were 6.78 times at increased risk of delivering LBW neonates compared to those who attended antenatal care (RRR:6.78,CI:2.39–19.25).

From the sociodemographic characteristics, mothers who resided in the urban area were at 47% lower risk of delivering LBW neonates than their rural counterparts (ARR: 0.53, CI: 0.32–0.9).

From the intermediate factors, first marriage was significantly associated with LBW for mothers who married after 18 years and above. Specifically, they were 41% less likely to deliver LBW neonate than mothers married before the age of 18 (RRR:0.59,CI:0.35–0.97), but marital age was not significantly associated with macrosomic birth (RRR:0.71,CI:0.4–1.23) (Table 3 **).**

## Discussion

In this study, we determined the incidence of LBW and macrosomia. We also identified the independent predictors of LBW and macrosomia on the same model.

Accordingly, we found the incidence of macrosomia to be 6.7%. The rate of macrosomia is within the worldwide and range of African studies [6]. This rate is higher than a prevalence from Democratic Republic Congo, Kenya, Niger, and Angola, but it is less than the rate from Algeria, Uganda, Nigeria, and most Asian countries [6]. This difference could be due to the nature of macrosomia trends, as it is expected to increase incrementally with the development of a country [6]. It is lower than the long-term Iranian cohort study and Nigerian retrospective study [11, 30]. In contrast to these studies, our study was conducted for a short period of time and was hospital-based, which may underestimate the incidence rate and may not indicate the true prevalence.

The incidence of LBW found was 10.5% which is higher than the reported rates in developed countries and lower than the rate of some sub-Saharan African studies [7, 31]. It is also lower than studies from northwest Ethiopia, Kenya and Zimbabwe [8–10]. The reason for this discrepancy may be the time this study is conducted and recent interventions in the prevention of LBW may have strengthen and contributed towards reduction in the incidence of LBW. Further this study was conducted in hospitals, which may indicate mothers with better socioeconomic status [16] and subsequently would underestimate the incidence rate. However, this rate is higher than the national retrospective household survey, which estimated 8.1% prevalence in the region, but in this national survey only 10.1% of mothers responded about birth weight [22].

### Predictors for macrosomia and LBW

Gestational age was one of the independent predictors of macrosomia and LBW. Post-maturity was significantly associated with macrosomia, which is similar to previous findings [6, 17]. This may be attributable to the period of post-maturity that there could lead to excessive fetal weight gain. Further, other potential causes may include maternal weight gain, diabetes, and other chronic disease [32, 33]. Prematurity is significantly associated with LBW and this finding is supported by previous findings from Kenya, Oman and Iran [10, 20, 34]. Similar to our study, in which we found (RRR: 15.4, CI: 9.18–25.9), the Iranian study also found a very strong association (OR: 42.82, 95% CI: 21.93–83.57). This showed that prematurity and LBW are strongly associated. Although it did not have a statistically significant association, we found over half 58% of LBW neonates were term births, indicating intrauterine growth retardation. It is evident that preterm births are exposed to LBW due to inadequate weight gain during pregnancy because of early delivery but intrauterine growth retardation demands further investigations.

Male neonates are also associated with increased risk of being macrosomic, which aligns with a previous meta-analysis conducted in 23 countries and a study in China [6, 18]. There may be biological reasons which could expose male neonates to excessive weight gain during pregnancy. Conversely, female birth is associated with being a LBW, which aligns with recent studies conducted in the African context [8–10].

Maternal complications were associated with macrosomia. This finding is supported with previous study for macrosomia [6, 17]. In our study, hemorrhage, pregnancy-induced hypertension, and obstructed labor were the major maternal complications which may be associated with macrosomia. High BMI of mothers was significantly associated with delivering macrosomic neonates, which concurs with previous studies which showed the association of obesity and high BMI with macrosomic birth [3, 18, 19]. As Ethiopia is a developing country, we are beginning to be affected by the double burden of diseases with emerging chronic disease like obesity (high BMI) [35], hence a parallel increase in macrosomia is expected.

Rural residence and early marriage were also associated with delivering LBW babies, which is a similar finding to previous studies [21, 31, 34, 36–38]. Rural mothers and those who were married young are at increased risk of delivering LBW often due to low risk awareness and are poor utilization of health services [23]. These gaps may expose these mothers to low quality of antenatal follow-up and related services. Further, these women may lack adequate nutrition due to their rural residence, in which most mothers in our study were farmers [39]. These factors could potentiate the incidence of LBW. In addition mothers who married early have immature body systems in which the body could not withstand the pregnancy and these situations expose the mothers to complications [40]. Large epidemiological studies also found increased risk for LBW with extreme of ages of mother [20, 21].

Having no antenatal follow up is significant predictors of LBW, which mirrors prior findings [8, 36, 38]. We had a few (1.8%) mothers who never had antenatal follow-up in our study and this widens the confidence interval. It is evident that mothers who never had antenatal follow up would be affected by delays in early detection and management of complications as well as not receiving supplements which could helpful to prevent prematurity and/or intrauterine growth retardation.

The strength of this data is that it is a prospective study. In addition, our study covers the entire range of hospitals in the study region so that it will be generalizable to all Ethiopian hospitals. This study concurrently considered both LBW and macrosomia and identified the common factors, such as gestational age and gender. It is favorable to tackle both of these problems at the same time.

The limitations of this study are that did not specifically study mothers with diabetes, or term births yielding low birth weight neonates, nor fully consider maternal complications owing to nature of our study. Further, this study was also conducted cross-sectionally and only in hospitals, whereby, in the study region, only 27% of mothers deliver in hospitals or another health care facility [28]. Consequently, this may underestimate and/or overestimate the rates.

## Conclusion

Our study revealed that mothers with early marriage, delivery of premature neonates, lacking antenatal follow-up, bearing female neonates, and/or who had pregnancy related complications are at higher risk to deliver low birth weight neonates. In addition, mothers who delivered male neonates, had pregnancy related complications, post-maturity, and high BMI are at risk of delivering macrosomic neonates. Therefore, strengthening antenatal follow-up, improving socioeconomic status and lifestyle, promoting early detection and management of complications, enhancing early detection of prematurity and post-maturity are recommended to prevent or mitigate both low birth weight and macrosomia and their long term consequences. We also recommend longitudinal studies on the long-term consequences of low birth weight and macrosomia throughout Ethiopia.

## Abbreviations

AIDS: Acquired immune deficiency syndrome
BMI: Body mass index
ETB: Ethiopian birr
HIV: Human immunodeficiency virus
LBW: Low birth weight
RRR: Relative risk ratio
WHO: World Health Organization
